# Complication Rates After TRUS Guided Transrectal Systematic and MRI-Targeted Prostate Biopsies in a High-Risk Region for Antibiotic Resistances

**DOI:** 10.3389/fsurg.2020.00007

**Published:** 2020-02-28

**Authors:** Mike Wenzel, Lena Theissen, Felix Preisser, Benedikt Lauer, Clarissa Wittler, Clara Humke, Boris Bodelle, Valentina Ilievski, Volkhard A. J. Kempf, Luis A. Kluth, Felix K. H. Chun, Philipp Mandel, Andreas Becker

**Affiliations:** ^1^Department of Urology, University Hospital Frankfurt, Frankfurt, Germany; ^2^University Hospital Frankfurt, Frankfurt, Germany; ^3^Department of Radiology, University Hospital Frankfurt, Frankfurt, Germany; ^4^Institute for Medical Microbiology and Infection Control, University Hospital Frankfurt, Frankfurt, Germany; ^5^University Center of Infectious Diseases, University Hospital Frankfurt, Frankfurt, Germany; ^6^University Center of Competence for Infection Control of the State of Hesse, Frankfurt, Germany

**Keywords:** transrectal prostate biopsy, complications, systematic biopsy, targeted biopsy, infection, biopsy naïve, repeat biopsy

## Abstract

**Introduction:** There is still an ongoing debate whether a transrectal ultrasound (TRUS) approach for prostate biopsies is associated with higher (infectious) complications rates compared to transperineal biopsies. This is especially of great interests in settings with elevated frequencies of multidrug resistant organisms (MDRO).

**Materials and Methods:** Between 01/2018 and 05/2019 230 patients underwent a TRUS-guided prostate biopsy at the department of Urology at University Hospital Frankfurt. Patients were followed up within the clinical routine that was not conducted earlier than 6 weeks after the biopsy. Among 230 biopsies, 180 patients took part in the follow-up. No patients were excluded. Patients were analyzed retrospectively regarding complications, infections and underlying infectious agents or needed interventions.

**Results:** Of all patients with follow up, 84 patients underwent a systematic biopsy (SB) and 96 a targeted biopsy (TB) after MRI of the prostate with additional SB. 74.8% of the patients were biopsy-naïve. The most frequent objective complications (classified by Clavien-Dindo) lasting longer than one day after biopsy were hematuria (17.9%, *n* = 32), hematospermia (13.9%, *n* = 25), rectal bleeding (2.8%, *n* = 5), and pain (2.2%, *n* = 4). Besides a known high MDRO prevalence in the Rhine-Main region, only one patient (0.6%) developed fever after biopsy. One patient each (0.6%) consulted a physician due to urinary retention, rectal bleeding or gross hematuria. There were no significant differences in complications seen between SB and SB + TB patients. The rate of patients who consulted a physician was significantly higher for patients with one or more prior biopsies compared to biopsy-naïve patients.

**Conclusion:** Complications after transrectal prostate biopsies are rare and often self-limiting. Infections were seen in <1% of all patients, regardless of an elevated local prevalence of MDROs. Severe complications (Clavien-Dindo ≥ IIIa) were only seen in 3 (1.7%) of the patients. Repeated biopsy is associated with higher complication rates in general.

## Introduction

Prostate cancer is still the most common cancer in men (1). It leads to the second most common cancer specific death in the United States and the third most in Europe (2, 3). To initiate a (curative) therapy, a histological cancer confirmation is needed. The gold standard of prostate cancer detection is a transrectal ultrasound (TRUS) guided biopsy (4). For TRUS guided prostate biopsy, a transrectal or transperineal approach is possible. Even though the cancer detection rates of both approaches are comparable, there is still an ongoing debate if a transrectal approach should be used in daily practice due to complication rates (5).

By the use of transrectal biopsy of the prostate, there is no need for general anesthesia, and it can be performed easily in an outpatient setting under antibiotic prophylaxis. On the other hand, transrectal biopsies can occur with rectal injuries and are suspected to go along higher infectious complications (6, 7).

Furthermore, with the implementation of the multiparametric MRI of the prostate (mpMRI) in daily usage for prostate cancer detection, the settings of prostate biopsies have changed. Recent studies have shown that the MRI targeted biopsy (TB) improves the detection rates for significant prostate cancer (≥ ISUP grade 2) compared to systematic biopsy (SB) (8, 9). Additionally, EAU Guidelines give a weak recommendation for a performance of mpMRI in biopsy naïve patients and strongly recommend a mpMRI for repeat biopsies (4). In most cases, SB is added to a TB, which has shown an increase in the numbers of cancer detection rates (10–12). This leads to an increased number of biopsy cores and also may increase the risk of complications of the biopsy.

As a consequence of developing antibiotic resistances in hospitals, complications after prostate biopsies are increasing (13). In both known approaches, haematuria, haematospermia, urinary retention, rectal bleeding, or prostatitis are the most common known complications (4, 14).

The objective of the present study was to analysis the complication rates of TRUS guided transrectal prostate biopsy and its correlation to the performance of SB alone or the combination of TB and SB. All biopsies have been performed in a tertiary care hospital located in a region with elevated prevalence of MDROs [e.g., vancomycin-resistant enterococci (VRE), methicillin-resistant Staphylococcus aureus (MRSA) and multidrug resistant gram negative bacteria (MDRGN)] with up to >90% resistance rates for penicillins, cephalosporins or flourquinolones in typical intestinal flora bacteria (unpublished data) (15–17). We assume that the combination of TB with SB yields to same incidence of complications rates than SB alone.

## Materials and Methods

### Study Population

After approval of the ethic committee, all 230 patients who underwent a TRUS guided transrectal biopsy of the prostate from 01/2018 to 05/2019 at the Department of Urology, University Hospital Frankfurt, Germany, were identified. Indications for performing a prostate biopsy were clinical parameters such as a suspicious digital rectal examination (DRE) or suspicious elevated PSA-levels. Moreover, patients with lesions ≥ PIRADS 3 in the inhouse or external mpMRT were recommended to undergo a prostate biopsy and included in this evaluation. All data were collected retrospectively.

### Antibiotic Prophylaxis Prior the Biopsy

All biopsies were taken under oral antibiotic prophylaxis with flourquinolones (levofloxacine 500 mg) in consistence with the EAU guidelines (4). The first dose was taken 1 day prior to biopsy and the second dose on the morning of the biopsy. The antibiotic prophylaxis was continued for 1–3 days after biopsy. Rectal swab culture or targeted antibiotic therapy was not performed as a standard prior to the biopsies.

### Biopsy Approach and Number of Cores

A transrectal approach by the use of local anesthesia was performed in all prostate biopsies. For local anesthesia, 10 ml bupivacaine was injected TRUS-guided as a periprostatic block on both sides (4, 18). For SB, 12-core biopsies were taken with 6 cores with a length of 15–22 mm from each prostate lobe. For TB, a high-end ultrasound machine HiVison (Hitachi Medical Systems) was used. A minimum of two cores, also with a length of 15–22 mm, was taken from each MRI-targeted lesion (≥ PIRADS 3). Additionally to the TB, SB was performed afterwards in the same biopsy session. Consequently, for TB and SB as a whole, a minimum of 14 cores were taken.

### Statistical Analysis

Within our clinical follow-up in the course of a standardized survey, complication rates were collected 6 weeks after the transrectal prostate biopsy at the earliest. Also, patients' baseline characteristics were collected from the specific patients' hospital files. The descriptive analysis contains proportions, frequencies, medians and interquartile ranges for category variables and was evaluated with the Mann-Whitney *U*-Test.

For the evaluation of statistical significance to a level of α = 5%, the Chi-square Test was used. Specifically, significant differences of complication rates after transrectal prostate biopsies between SB against TB + SB were tested. Additionally, biopsy-naïve patients against repeat biopsies were investigated for significant differences in complication rates. Furthermore, risk stratifications for the occurrence of complications were done.

For statistical analysis, the software R statistics (version 3.4.4) was used.

## Results

Among the 230 patients, 180 (78.3%) took part in the clinical follow up (Table 1). Of these 180 patients, 84 underwent a SB alone, and 96 underwent a TB + SB after the prior MRI of the prostate.

**Table 1:** Baseline characteristics for patients with transrectal prostate biopsy at University Hospital Frankfurt between 01/2018 and 05/2019 stratified for SB and SB + TB.

**Variable**		**Overall**	**SB**	**SB + TB**	***p*-value**
Patients with transrectal biopsy, n		230	105	125	
Patients with clinical follow up, n (%)		180 (78.3)	84 (80)	96 (76.8)	
Age at biopsy, median (IQR)		66 (61–72)	66 (61–72.2)	66 (59–71)	0.164
PSA (ng/ml), median (IQR)		7.8 (5.3–14.7)	10 (6.2–46.5)	6.9 (4.8–10.4)	0.178
Prostate volume (ccm), median (IQR)		45 (35–64.5)	45 (37–70)	40 (35–60)	0.072
DRE suspicous for PCA, n (%)		69 (30)	37 (35.2)	32 (25.6)	0.149
Previous biopsies, n (%):	0	172 (74.8)	86 (81.9)	86 (68.8)	0.025
	1	41 (17.8)	11 (10.5)	30 (24)	
	>2	15 (6.5)	6 (5.7)	9 (7.2)	
Cores per biopsy, median (IQR)		14 (12–17)	12 (12–12)	17 (14–20)	<0.01
Positive Cores per biopsy, median (IQR)		3 (1–7)	3 (1–6)	3 (1–7)	0.91
If PCA: ISUP Grade, n (%):	GG1	19 (8.3)	10 (9.5)	9 (7.2)	0.032
	GG2	35 (15.2)	10 (9.5)	25 (20)	
	GG3	33 (14.3)	11 (10.5)	22 (17.6)	
	GG4	19 (8.3)	10 (9.5)	9 (7.2)	
	GG5	39 (17)	25 (23.8)	14 (11.2)	
	noPCA	85 (37)	39 (37.1)	46 (36.8)	

*SB, systemic biopsy; TB, targeted biopsy; IQR, interquartile range; PSA, prostate specific antigen; DRE, digital rectal examination; PCA, Prostate cancer; GG, Grade Group*.

The median age of the patients was 66 years. The overall median PSA-level was 7.8 ng/ml [interquartile range (IQR) 5.3–14.7]. While the median PSA-level in the SB group was 10 ng/ml (IQR 6.2–46.5). the median PSA-level was 6.9 ng/ml (IQR 4.8–10.4) (*p* < 0.01) in the SB + TB group.

One hundred seventy-two men (74.8%) were biopsy-naïve, 41 (17.8%) had one and 15 (6.5%) had two or more prior prostate biopsies done (2 patients with unknown prior biopsies, 0.9%). The median prostate volume was 45 ccm (IQR 35–64.5), measured through transrectal ultrasound. For patients within the SB cohort, the median number of cores which were taken was 12 (IQR 12–12) compared to the SB + TB cohort with a median number of 17 cores (IQR 14–20) (*p* < 0.01).

From the 180 patients which took part in our clinical follow-up, 163 (90.6%) would favor the transrectal approach with periprostatic anesthesia for their prostate biopsy, if they had to make the choice again. Twelve (6.7%) patients would choose another approach or setting for a repeat prostate biopsy and two (1.1%) patients were undecided. No significant differences were seen between the SB and SB + TB cohort (91.7 and 89.6% would choose the transrectal biopsy approach again). 93.4 % of biopsy-naïve patients would favor transrectal prostate biopsy again, compared to 81.4% in patients with at least one prior biopsy (*p* < 0.05).

One hundred sixty-one patients (89.4%) reported within the clinical follow-up that they had no subjectively perceived complaints after the transrectal prostate biopsy (Table 2). No significant differences between the SB vs. TB + SB and biopsy-naïve vs. repeat-biopsy cohorts (90.5 vs. 88.5% and 99 vs. 93.3%) were seen.

**Table 2:** Complications after transrectal prostate biopsy of patients between 01/2018 and 05/2019 stratified for SB and SB + TB.

**Variable**		**Overall**	**SB**	**TB + SB**	***p*-value**
Patients subjectively reported about complications after biopsy, n (%):	No	161 (89.4)	76 (90.5)	85 (88.5)	0.631
	Yes	16 (8.9)	6 (7.1)	10 (10.4)	
	Undecided	3 (1.7)	2 (2.4)	1 (1.1)	
Patient would choose the same approach in another biopsy, n (%):	Yes	163 (90.6)	77 (91.7)	86 (89.6)	0.947
	No	12 (6.7)	5 (6)	7 (7.3)	
	Undecided	2 (1.1)	1 (1.2)	1 (1)	
	Unknown	3 (1.7)	1 (1.2)	2 (2.1)	
Gross hematuria (in days after biopsy), n (%)	0–1	148 (82.2)	69 (82.1)	79 (82.3)	0.532
	2–5	12 (6.7)	4 (4.8)	8 (8.3)	0
	5–10	9 (5)	4 (4.8)	5 (5.2)	0
	>14	11 (6.2)	7 (8.4)	4 (4.1)	0
Hematospermia (in days after biopsy), n (%)	0–1	155 (86.1)	73 (86.9)	82 (85.4)	0.942
	>1	25 (13.9)	11 (13.1)	14 (14.6)	
Rectal bleeding (in days after biopsy), n (%)	0–1	175 (97.2)	82 (97.6)	93 (96.9)	0.999
	2–3	4 (2.3)	1 (1.2)	3 (3.1)	0.172
	>14	1 (0.6)	1 (1.2)	(0)	
Sacral/gluteal pain, n (%)	No	176 (97.8)	84 (100)	92 (95.8)	0.166
	Yes	4 (2.2)	0 (0)	4 (4.2)	
Fever after biopsy, n (%)		1 (0.6)	1 (1.2)	0 (0)	0.317
Physician consultation after biopsy? n (%)	No	176 (97.6)	82 (97.6)	94 (97.9)	0.999
	Yes	4 (2.4)	2 (2.4)	2 (2.1)	
Reason of consultation, n (%)	Hematuria	1 (0.6)	0 (0)	1 (1)	0.401
	Rectal bleeding	1 (0.6)	0 (0)	1 (1)	
	Urinary retention	1 (0.6)	1 (1.2)	0 (0)	
	Unknown	1 (0.6)	1 (1.2)	0 (0)	

*SB, systemic biopsy; TB, targeted biopsy*.

After evaluation of objective complication criteria, the most frequent complications were hematuria, hematospermia, rectal bleeding, and discomfort or pain in the sacral/gluteal region. One hundred forty-eight (82.2%) of the patients reported about no or 1 day of gross hematuria after biopsy. Thirty-two patients (17.8%) had gross hematuria longer than 1 day, of which 11 patients (6.2%) had gross hematuria for longer than 14 days. 155 (86.1.%) patients reported about no or only short lasting (up to 1 day) hematospermia. Furthermore, 175 patients (97.2%) had no or 1 day of rectal bleeding after biopsy. 5 patients (2.8%) indicated rectal bleeding longer than 1 day after biopsy. For gross hematuria, hematospermia and rectal bleeding, no significant differences in the SB vs. TB + SB and biopsy-naïve vs. repeat-biopsy cohorts were seen. After the biopsy four patients (2.2%) reported about sacral/gluteal pain.

One patient (0.6%) developed fever after transrectal prostate biopsy. Four (2.4%) patients consulted a physician after transrectal prostate biopsy due to complications. Reasons were necessary bladder irrigation due to hematuria (1 patient; 0.6%, Clavien-Dindo IIIa), urinary retention (1 patient; 0.6%, Clavien-Dindo IIIa), rectal bleeding with need of hemostasis (1 patient; 0.6%, Clavien-Dindo IIIb), as well as one unknown reason. The consultation of a physician was significantly higher in the group of repeat-biopsies compared to biopsy-naïve patients (*p* < 0.05) (Table 3). Severe complications (Clavien-Dindo ≥IIIa) was only seen in 3 (1.7%) of the patients, without significant differences between the two compared groups.

**Table 3:** Complications after transrectal prostate biopsy of patients between 01/2018 and 05/2019 stratified for repeat biopsies and biopsy-naïve patients.

**Variable**		**Overall**	**Repeat biopsy (≥1)**	**Biopsy-naive**	***p*-value**
Patients subjectively reported about complications after biopsy, n (%):	No	161 (89.4)	36 (83.7)	125 (91.2)	0.622
	Yes	16 (8.9)	5 (11.6)	11 (8)	
	Undecided	3 (1.7)	2 (4.7)	1 (0.8)	
Patient would choose the same approach in another biopsy, n (%):	Yes	163 (90.6)	35 (81.4)	128 (93.4)	0.005
	No	12 (6.7)	5 (11.6)	7 (5.1)	
	Undecided	2 (1.1)	0 (0)	2 (1.5)	
	Unknown	3 (1.7)	3 (7)	0 (0)	
Gross hematuria (in days after biopsy), n (%)	0–1	148 (82.2)	38 (88.4)	110 (80.3)	0.326
	2–5	12 6.7)	3 (7.0)	9 (6.6)	
	5–10	9 (5.0)	1 (2.3)	8 (5.8)	
	>14	11 (6.2)	1 (2.3)	10 (7.2)	
Hematospermia (in days after biopsy), n (%)	0–1	155 (86.1)	39 (90.7)	116 (84.7)	0.457
	>1	25 (13.9)	4 (9.3)	21 (15.3)	
Rectal bleeding (in days after biopsy), n (%)	0–1	175 (97.2)	42 (97.7)	133 (97.1)	0.999
	2–3	4 (2.3)	1 (2.3)	2 (2.2)	0.841
	>14	1 (0.6)	0 (0)	1 (0.7)	
Sacral/gluteal pain, n (%)	No	176 (97.8)	42 (97.7)	134 (97.8)	1
	Yes	4 (2.2)	1 (2.3)	3 (2.2)	
Fever after biopsy, n (%)		1 (0.6)	1 (2.3)	0 (0)	0.317
Physician consultation after biopsy? n (%)	No	176 (97.6)	40 (93)	136 (99.3)	0.04
	Yes	4 (2.4)	3 (7)	1 (0.7)	
Reason of consultation, n (%)	Hematuria	1 (0.6)	1 (2.3)	0 (0)	
	Rectal bleeding	1 (0.6)	1 (2.3)	0 (0)	
	Urinary retention	1 (0.6)	0 (0)	1 (0.7)	
	Unknown	1 (0.6)	1 (2.3)	0 (0)	

*SB, systemic biopsy; TB, targeted biopsy*.

## Discussion

Transrectal and transperineal approaches are both safe and commonly used techniques for prostate cancer detection through prostate biopsy. The debate whether a transrectal or a transperineal approach should be performed as a gold standard with special attention to the side effects and economic reasons is still on (4, 5, 14, 19). To evaluate the risks of transrectal TRUS-guided prostate biopsy at a university hospital with high prevalence of multidrug-resistant bacteria/organisms, complication rates were examined. Also, risk stratification for SB, TB + SB, biopsy-naïve patients as well as repeat biopsies were done. For the retrospective analyses, we included all patients who underwent a transrectal prostate biopsy with antibiotic prophylaxis of flourquinolones between 01/2018 and 05/2019 at University Hospital Frankfurt until the European Medicine Agency (EMA) published their recommendation for the restrictive use of flourquinolones regarding potentially permanent side effects.

First, the prevention of serious infectious complications is often seen as an argument for a transperineal approach for prostate biopsies. Also, an increasing risk for transrectal biopsy-related complications was described and published recently (6, 7, 13, 20, 21). Rates of fever, prostatitis and epididymitis after transrectal biopsy are mentioned in the literature with 3.5, 1.0, and 0.7%, respectively (4, 22, 23). Hospitalization due to serious infections is quite rare but seen with ~1.0–2.8% of all patients (13, 20, 24, 25). In our data, developing fever after transrectal prostate biopsy was seen just in one patient (0.6%) without the need of emergency treatment or clinical admission of these patient. Thus, it could be followed that urine or rectal swab analyses is not necessary to be performed prior every prostate biopsy without any high-risk situation (e.g., history of prior prostatitis or genitourinary infection), even in areas of known high prevalence of antibiotic resistances.

Furthermore, the consultation of a physician after transrectal prostate biopsy was reported just in four cases (2.4%) of all biopsies. Reasons were hematuria with need for bladder irrigation, urinary retention (both Clavien-Dindo Grade IIIa), rectal bleeding with need of surgical intervention (Clavien-Dindo Grade IIIb) and one unknown consequence. This data also goes in line with previous reports that have shown an incidence of 0.4–0.8% for urinary retention after transrectal prostate biopsy (22, 26). No significant differences were seen in our data between the SB and SB + TB group regarding infectious complications or consulting a physician in an emergency due to hematuria, rectal bleeding, or urinary retention. Consequently, our data clearly underlines that SB as well as SB + TB with a transrectal approach can be performed safely without serious infectious complications, even though high rates of multiresistant bacterial species (15–17).

Second, side effects after transrectal prostate biopsy are quite rare. Overall, no complications were subjectively reported by 89.4% of our patients. The most common side effects of the transrectal prostate biopsy approach are gross hematuria, hematospermia und rectal bleeding. None of these side effects lasting for more than 1 day after the biopsy was seen in 82.2, 86.1, and 97.2% of our patients, respectively. Gross hematuria for longer than 14 days was reported in 6.2% of our patients. These rates are quite lower than reported in the prospective trial of Rosario et al. who showed a hematuria rate of 20% 14 days after biopsy. One possible explanation might be the retrospective setting of our analyses (26). Also, due to Patient education prior to biopsy, a few patients did not subjectively see short term gross hematuria as a relevant biopsy related complication.

Further, our hematospermia rates of patients who were suffering for longer than 1 day (2.8%) are lower than reported in other studies where 50.4% of the patients suffered for longer than 3 days (24). The range of any hematospermia after transrectal prostate biopsy varies between 1.1 and 92.6% in the literature (13, 22, 26–28).

Also, the prospective data from Rosario et al. showed that rectal bleeding after transrectal prostate biopsy is quite common but often self-limiting and seen as a major problem for just 2.5% of all patients (26). Additionally, rectal bleeding rates during the first days after the biopsy are given with <2.2% in the EAU guidelines (4). These results are in line with our rates of 2.3% of rectal bleeding for 2–3 days and 0.6% longer than 14 days after transrectal prostate biopsy.

In addition, pain of the gluteal or sacral region was reported quite often after transrectal prostate biopsy. Serious pain events were reported in 5.7% within 7 days after biopsy (26). In our cohort, just 2.2% of our patients reported about any kind of pain during our clinical follow-up after transrectal biopsy. Comparing non-infectious complication rates, same low rates could also be expected in a transperineal approach (29).

Third, there is still a lack of information if the number of cores taken during prostate biopsies influences the risk for complications afterwards: The prospective trial of Ghani et al. investigated a higher risk for rectal bleeding if more than six cores were taken and Chowdhury et al. saw a significantly higher risk for all bleeding complications with an increasing number of cores, whereas Berger et al. found no differences between six-, ten- and fifteen-core prostate biopsies (28, 30, 31). In our analysis, the median cores per biopsy were 12 in the SB group and significantly different from 17 cores in median in the SB + TB group. In all analyses, no significant differences were seen in our data between these two groups regardless of any complication of hematuria, hematospermia, rectal bleeding or sacral/gluteal pain.

Fourth, previous publications reported that repeat biopsies are an independent risk factor for complications after transrectal prostate biopsy compared to biopsy-naïve patients (32). Also, EAU guidelines list previous biopsies as a factor for inducing antibiotic quinolone resistance and post-biopsy infections (4). Loeb et al. found an ~1.7 higher risk for hospitalization and infectious complications with every repeat biopsy. Additionally, the risk for serious complications was increased by 2.2 times (33). Within our analyses, we also found a significantly higher rate of a consultation of a physician due to subjective discomforts after transrectal prostate biopsy in the cohort of men with repeat biopsies compared to biopsy-naïve patients (7 vs. 0.7%) (*p* < 0.05). These results clearly demonstrate the need of raising awareness before recommending and performing a repeat biopsy.

The present study has several limitations. First and foremost, our manuscript is based on a retrospective analysis. Namely, all data were collected 6 weeks after the transrectal prostate biopsy at the earliest at one tertiary care center. This may lead to unknown or not detailed reported complications in the data analyses. Moreover, the possibility of a selection bias exists with the clinical follow up of the 180 patients out of 230 biopsies. Further, all biopsies were performed under antibiotic prophylaxis with flourquinolones, without any prior MDRO screening. Until now, it is not clearly demonstrated if complication rates after transrectal prostate biopsy differ with other antibiotic regimes after EMA flourquinolone restrictions. Finally, there is no comparison to a group with transperineal biopsy approach.

Taken together, our analyses demonstrate that the transrectal approach for prostate biopsy is a safe approach with low complication rates which is a well-tolerated and accepted by the patients. Finally, it can be concluded that despite increasing and high incidences of multiresistant bacterial colonies, transrectal prostate biopsy can be performed easily and safely. Our study cohort provides a contemporary, much needed update compared to previous studies with either limited patient numbers or historical cohorts, since biopsy strategies, number of taken cores, and targeted biopsy due to the improvement of MRI have changed.

## Data Availability Statement

All datasets generated for this study are included in the article.

## Ethics Statement

The study was approved by the institutional review boards of the University Cancer Centre Frankfurt and the Ethical Committee at the University Hospital Frankfurt.

## Author Contributions

MW: protocol development, acquisition of the analyzed data, analysis and interpretation of data, substantial contributions to the conception of work, drafting the work, and revising the work for the intellectual content. CH and LT: protocol development, acquisition of the analyzed data. FP: data analysis. BL, CW, VI, VK, LK, BB, and PM: acquisition of analyzed data, contribution to the conception of work, final approval of manuscript. FC: substantial contributions to the conception of the work, critical revision of intellectual content. AB: substantial contributions to the conception of work, critical revision of work, data analysis, and final approval of the version to be published. All authors complied with all aspects of the work. They ensure that questions related to the accuracy of the work are adequately discussed and solved.

### Conflict of Interest

The authors declare that the research was conducted in the absence of any commercial or financial relationships that could be construed as a potential conflict of interest.
